# What is the effect of ischemic preconditioning on the kinetics of pulmonary oxygen uptake and muscle deoxygenation during exercise?

**DOI:** 10.14814/phy2.12540

**Published:** 2015-09-04

**Authors:** Jeann L C Sabino-Carvalho, Thales Coelho Barbosa, Bruno Moreira Silva

**Affiliations:** 1 Postgraduate Program in Translational Medicine, Federal University of São PauloSão Paulo, Brazil; 2 Department of Physiology and Pharmacology, Fluminense Federal UniversityNiterói, Rio de Janeiro, Brazil; 3 Department of Physiology, Federal University of São PauloSão Paulo, Brazil

Ischemic preconditioning (IPC, brief cycles of ischemia) induces protection against ischemia–reperfusion injury, due to IPC-mediated change in the metabolism of skeletal muscles and micro- and macrovascular function. Since high-intensity aerobic exercise depends on such physiological factors, it has been hypothesized that IPC can also improve exercise performance. Although some studies confirmed this hypothesis (Salvador et al. 2015), others did not (Salvador et al. 2015). Moreover, even when IPC improved exercise performance, the mechanisms involved were unclear. For example, increase in maximal oxygen consumption (V̇O_2max_) (De Groot et al. 2010) and decrease in blood lactate concentration (Bailey et al. 2012) have been reported, but these findings were not unanimous among studies (Salvador et al. 2015).

In this context, a recent study by Kido et al. (2015) examined the effects of IPC of the lower limbs on the kinetics of pulmonary V̇O_2_ and muscle deoxygenation during square-wave transitions from low- to moderate-intensity and from moderate- to severe-intensity cycling exercise. Besides, authors assessed the effect of IPC on the time to exhaustion in the severe-intensity workload. Authors found that IPC increased the time to exhaustion, which supports an increase in exercise performance. Furthermore, IPC did not change the pulmonary V̇O_2_ kinetics, but changed the kinetics of muscle deoxygenation. These findings are original and advance the knowledge about the effect of IPC on exercise physiology. However, some aspects of the experimental protocol and data analysis deserve attention to interpret these findings.

Authors used just one exercise transition to calculate the pulmonary V̇O_2_ kinetics. Nevertheless, repetitions of exercise transitions are crucial to increase the signal-to-noise ratio. This was proposed by Lamarra et al. (1987), who described the following equation to estimate the confidence interval (Kn) of the time constant:

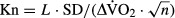
 where *L* is a constant that depends on the value of the underlying time constant; SD is the standard deviation of the breath-by-breath fluctuation in V̇O_2_; ΔV̇O_2_ is the V̇O_2_ amplitude above the baseline level; and *n* is the number of exercise transitions. Based on this equation, typically, four to eight exercise bouts are used for moderate-intensity transitions and two to four exercise bouts are used for severe-intensity transitions (Poole and Jones 2012). Thus, the use of just one exercise transition increased the error to calculate kinetics parameters, which may have concealed an effect of IPC on V̇O_2_ kinetics.

Despite the fact that authors did not find an effect of IPC on the kinetics of pulmonary V̇O_2_, the kinetics of muscle deoxygenation was changed by IPC. Muscle deoxygenation, as measured by near-infrared spectroscopy, is a proxy of muscle oxygen extraction. Thus, a change in this variable should influence the pulmonary V̇O_2_ kinetics, which was not observed, maybe due to the aforementioned limitation on the V̇O_2_ kinetics analysis. Noteworthy, authors investigated the effect of IPC on work-to-work transitions, and it is known that the phase II of pulmonary V̇O_2_ kinetics is slowed when high-intensity exercise is initiated from a moderate-intensity work rate in comparison with a rest-to-work transition (Dimenna et al. 2010). This has been attributed to the fact that the muscle fibers are already active, and then cellular respiration adapts more slowly to the transition in workload (Dimenna et al. 2010). Thus, it is unknown if the effect of IPC would be different in rest-to-work than work-to-work transitions.

Specifically regarding the muscle deoxygenation, authors concluded that IPC accelerated its kinetics in the transition from low- to moderate-intensity exercise, but did not change the kinetics in moderate- to severe-intensity exercise. Moreover, the amplitude of the deoxy-Hb/Mb response in the low- to moderate-intensity exercise was reduced by IPC, whereas IPC did not change the amplitude in the moderate- to severe-intensity exercise. However, as the amplitude of the deoxy-Hb/Mb was reduced in the low- to moderate-intensity exercise, the baseline value for the moderate- to severe-intensity exercise was consequently reduced, which may have compromised the interpretation about the kinetics of deoxy-Hb/Mb in the moderate- to severe-intensity transition. In addition, the amplitude of deoxy-Hb/Mb response during exercise was analyzed relative to the plateau value during a 10-min arterial occlusion. But, authors did not report the values obtained during this maneuver. Were these values affected by the IPC? If these values were affect, does it explain the difference in amplitude during exercise? Alternatively, if data were normalized by the resting baseline value, would the interpretation be similar? At last, the time delay, time constant, and mean response time were calculated from data normalized by the end exercise amplitude. Would the interpretation be similar if the analysis was done from data normalized by the resting baseline value or by the plateau during the 10-min arterial occlusion?

Besides Kido et al. (2015), two studies have also investigated the IPC effect on the balance between O_2_ utilization and delivery in the microcirculation via near-infrared spectroscopy. First, Barbosa et al. (2015) studied the effect of the IPC of both legs on muscle deoxygenation and time to task failure in a constant load rhythmic handgrip exercise. In this study, the improvement of exercise tolerance was accompanied by higher deoxy-Hb/Mb at peak exercise compared to control. Patterson et al. (2015) then reported greater tissue oxygenation index (i.e., lower deoxygenation) during cycling sprints, when sprints were preceded by IPC of the legs. Summing up, the effect of IPC on muscle deoxygenation found by Kido et al. (2015) and Patterson et al. (2015) (i.e., lower deoxygenation) differ from Barbosa et al. (2015) (i.e., higher deoxygenation). Thus, the effect of IPC on muscle deoxygenation and on other mechanisms during exercise still needs clarification. More importantly, it is impossible to blind the subjects about the IPC exposure and all previous studies have not circumvented this problem properly (Salvador et al. 2015). Thus, future studies should rigorously avoid the placebo effect on the investigation of the ergogenic effect of IPC (e.g., comparing the IPC with a placebo pill or other placebo interventions), since it may partially explain the reported improvement in exercise performance (Marocolo et al. 2015).
